# Retracted: Correlation of Serum IL-18, BDNF, and IL-1*β* with Depression and Prognosis after Acute Exacerbation of Chronic Obstructive Pulmonary Disease

**DOI:** 10.1155/2022/9893716

**Published:** 2022-12-04

**Authors:** Computational and Mathematical Methods in Medicine

*Computational and Mathematical Methods in Medicine* has retracted the article titled “Correlation of Serum IL-18, BDNF, and IL-1*β* with Depression and Prognosis After Acute Exacerbation of Chronic Obstructive Pulmonary Disease” [1] due to concerns that the peer review process has been compromised.

Following an investigation conducted by the Hindawi Research Integrity team [2], significant concerns were identified with the peer reviewers assigned to this article; the investigation has concluded that the peer review process was compromised. We therefore can no longer trust the peer review process and the article is being retracted with the agreement of the Chief Editor.
